# Effect of Epirubicin Plus Paclitaxel vs Epirubicin and Cyclophosphamide Followed by Paclitaxel on Disease-Free Survival Among Patients With Operable *ERBB2*-Negative and Lymph Node–Positive Breast Cancer: A Randomized Clinical Trial

**DOI:** 10.1001/jamanetworkopen.2023.0122

**Published:** 2023-02-24

**Authors:** Peng Yuan, Yikun Kang, Fei Ma, Ying Fan, Jiayu Wang, Xue Wang, Jian Yue, Yang Luo, Pin Zhang, Qing Li, Binghe Xu

**Affiliations:** 1 Department of VIP Medical Oncology, National Cancer Center, National Clinical Research Center for Cancer, Cancer Hospital, Chinese Academy of Medical Sciences and Peking Union Medical College, Beijing, China; 2 Department of Medical Oncology, National Cancer Center, National Clinical Research Center for Cancer, Cancer Hospital, Chinese Academy of Medical Sciences and Peking Union Medical College, Beijing, China

## Abstract

**Importance:**

Adjuvant therapy is an important and effective treatment for breast cancer. However, there is a lack of head-to-head clinical trials comparing the regimens epirubicin plus paclitaxel (EP) vs epirubicin and cyclophosphamide followed by paclitaxel (EC-P) in breast cancer.

**Objective:**

To evaluate the noninferiority of a cyclophosphamide-free (EP) regimen compared with the standard EC-P regimen for patients with operable hormone receptor–positive, *ERBB2* (formerly *HER2*)-negative, lymph node–positive breast cancer.

**Design, Setting, and Participants:**

This prospective, open-label, phase 3, noninferiority randomized clinical trial was conducted from June 1, 2010, to June 30, 2016, in the Cancer Hospital, Chinese Academy of Medical Sciences, Beijing. Patients with hormone receptor–positive, *ERBB2*-negative, lymph node–positive operable breast cancer were included and randomized into 2 treatment groups. Data were analyzed from June 30, 2016, to November 1, 2022.

**Interventions:**

Patients received adjuvant epirubicin (75 mg/m^2^) and paclitaxel (175 mg/m^2^) every 3 weeks for 6 cycles (EP regimen) or epirubicin (90 mg/m^2^) and cyclophosphamide (600 mg/m^2^) every 3 weeks for 4 cycles followed by paclitaxel (175 mg/m^2^) every 3 weeks for 4 cycles (EC-P regimen) as the intention-to-treat (ITT) population.

**Main Outcomes and Measures:**

The primary outcome was disease-free survival (DFS), and the secondary outcomes included overall survival (OS), distant DFS, and safety.

**Results:**

A total of 900 patients were registered, and 813 eligible patients (median age, 48 [IQR, 41-56] years) were randomly assigned to the EP group (n = 407) or the EC-P group (n = 406) after the surgical procedure. Through a median follow-up of 93.6 (IQR, 60.9-114.1) months, the hazard ratio (HR) of DFS for EP vs EC-P was 0.82 (95% CI, 0.62-1.10; 5-year DFS, 86.0% vs 80.6%; noninferior *P* = .001). The 5-year OS for the ITT population treated with the EP or the EC-P regimen was 94.7% vs 95.0%, respectively (HR, 0.95 [95% CI, 0.61-1.49]). Patients in the EP group had more frequent toxic effect events than those in the EC-P group.

**Conclusions and Relevance:**

In this prospective, open-label, phase 3, randomized clinical trial, the EP regimen was noninferior to the EC-P regimen. These findings supported that the EP regimen could be an effective adjuvant chemotherapy regimen for women with *ERBB2*-negative breast cancer.

**Trial Registration:**

ClinicalTrials.gov Identifier: NCT01134523

## Key Points

**Question:**

Is long-term efficacy of adjuvant regimen epirubicin plus paclitaxel (EP) noninferior to the standard regimen epirubicin and cyclophosphamide followed by paclitaxel (EC-P) in operable *ERBB2*-negative, lymph node–positive breast cancer?

**Findings:**

In this phase 3 randomized clinical trial of 813 patients with a median follow-up of 94 months, 5-year disease-free survival for patients receiving EP and EC-P was 86% and 81%, respectively. The 5-year overall survival was 95% and 95%, respectively.

**Meaning:**

In this study, the EP regimen was noninferior to the EC-P regimen and was an effective adjuvant chemotherapy regimen for women with *ERBB2*-negative breast cancer.

## Teaser

This randomized clinical trial compares the long-term efficacy and toxic effects of epirubicin plus paclitaxel and the standard epirubicin and cyclophosphamide followed by paclitaxel regimen among women with *ERBB2*-negative, lymph node–positive breast cancer.

## Introduction

Breast cancer is the most commonly diagnosed cancer (11.7% of total cases) in the world and is the leading cause of cancer-related death in women.^1,2^ In the US, mortality due to breast cancer declined from 2% to 3% annually during the 1990s and 2000s to 1% annually from 2013 to 2019, reflecting the efficacy of early screening and adjuvant chemotherapy in recent years.^3^

Based on the major prospective clinical trials,^4,5^ there are several standard chemotherapy options, typically containing both an anthracycline and a taxane. Doxorubicin and cyclophosphamide for 4 cycles followed by paclitaxel for 4 cycles (EC-P regimen) is a common regimen.^6^ Dose-dense EC-P given every 2 weeks with growth factor support after each chemotherapy cycle is superior to an older schedule of every 3 weeks.^7^ Other optimal schedules of an anthracycline followed by a taxane include weekly paclitaxel for 12 weeks or docetaxel every 3 weeks for 4 cycles.^8,9^ However, the treatment duration of the EC-P regimen is long, which affects the initiation and efficacy of radiotherapy. The dose-dense EC-P regimen also has serious adverse effects, and the quality of life of those patients is significantly decreased. In addition, application of cyclophosphamide may lead to amenorrhea and premature senescence, affecting the reproductive function of young women.

Actually, the epirubicin plus paclitaxel (EP) regimen also reached a satisfactory efficacy for metastatic and locally advanced breast cancer, with good tolerability.^10^ However, head-to-head clinical trials comparing the regimens EC-P and EP are lacking. Therefore, we compared the long-term efficacy and toxic effects of the EC-P and EP regimens to provide an evidence-based medical basis for the selection of the chemotherapy regimen for the clinical treatment of middle- to high-risk breast cancer with positive axillary lymph nodes.

## Methods

### Study Design

This prospective, open-label, phase 3, noninferiority randomized clinical trial of patients with *ERBB2* (formerly *HER2*)-negative operable breast cancer was approved by the institutional ethics committee of the Cancer Hospital, Chinese Academy of Medical Sciences. The trial was conducted according to the International Conference on Harmonization Good Clinical Practice guidelines and ethical principles in the Declaration of Helsinki.^11^ All patients were required to sign an informed consent form before enrollment and randomization. The results were reported according to the Consolidated Standards of Reporting Trials (CONSORT) reporting guideline.

The trial was designed as a 2-group prospective trial to test the noninferiority of a cyclophosphamide-free regimen including epirubicin (75 mg/m^2^) and paclitaxel (175 mg/m^2^) every 3 weeks for 6 cycles (EP regimen), compared with epirubicin (90 mg/m^2^) and cyclophosphamide (600 mg/m^2^) every 3 weeks for 4 cycles followed by paclitaxel (175 mg/m^2^) every 3 weeks for 4 cycles (EC-P regimen) in patients with hormone receptor–positive *ERBB2*-negative lymph node–positive operable breast cancer. Assignment to the treatment groups was randomized with a ratio of 1:1. The trial protocol is provided in Supplement 1.

### Study Population

Eligible patients were women with histologically confirmed, operable primary invasive breast cancer with known hormone receptor status, *ERBB2*-negative status, and no evidence of metastatic disease by standard laboratory and radiological examination results. Inclusion criteria were (1) being older than 18 years; (2) having node-positive tumors; (3) having hormone receptor–positive, *ERBB2*-negative status; (4) having a Karnofsky score of 70 or greater; and (5) being within 6 weeks after the surgery. Patients who had received neoadjuvant therapy (including chemotherapy, radiotherapy, or endocrine therapy) were excluded. Patients with serious active infections, severe organ dysfunction, left ventricular ejection fraction less than 50%, myocardial infarction, pregnancy, lactation, or Eastern Cooperative Oncology Group performance status 2 or greater were excluded. On completion of treatment, patients underwent follow-up surveillance and were scheduled to be seen every 3 months for the first 2 years and every 6 months after that for 10 years.

Chemotherapy was administered before radiotherapy if radiotherapy was indicated. Radiotherapy was completed by patients who received breast conservation or with 4 or more involved axillary lymph nodes or those with 1 to 3 involved axillary lymph nodes along with other high-risk factors. On completion of chemotherapy and/or radiotherapy, endocrine therapy (the regimen was decided by the physicians) was administered to patients for 5 years.

### Outcomes

The primary outcome was disease-free survival (DFS), defined as the time from randomization to occurrence of a new event, including local recurrence, regional relapse, distant metastasis, or death from any cause (excluding a second nonbreast invasive cancer). Patients alive without any predefined event were censored at the time of the last follow-up. The secondary outcomes included (1) overall survival (OS), defined as the time from randomization to death from any cause; (2) distant DFS (DDFS), defined as the time from randomization to the earliest distant metastasis or death from any cause; (3) DFS-s, defined as the time from randomization to occurrence of a new event, including local recurrence, regional relapse, distant metastasis, and second nonbreast invasive cancer; and (4) safety, which was assessed throughout the study treatment according to the Common Terminology Criteria for Adverse Events, version 4.0.

### Statistical Analysis

Data were analyzed from June 30, 2016, to November 1, 2022. This trial was designed to evaluate the noninferiority of the EP vs EC-P regimens. The test was designed with 80% power at the 1-sided α of .05. The trial assumed a 5-year DFS of 83% for the EC-P regimen.^12,13^ Noninferiority was defined as the 5-year DFS of the EP regimen being no worse than an absolute value of 5% below the EC-P regimen, with a limiting hazard ratio (HR) of 1.30. Under these assumptions, the sample size was approximately 800 patients, with a ratio of 1:1 in each group. The HRs were obtained using the Cox proportional hazards regression model. Noninferior *P* values were calculated according to a previous study.^14^

The statistical analysis was performed using SPSS software, version 23.0 (IBM Corporation), and the GraphPad Prism program, version 7.0 (GraphPad Software). All efficacy analyses were performed in the intention-to-treat (ITT) population. We used the χ^2^ test or the nonparametric Wilcoxon-Mann-Whitney test to compare the outcomes between the 2 groups. We calculated DFS, OS, and DFS-s using the Kaplan-Meier method and analyzed them using the stratified log-rank test. We obtained HRs with 95% CIs using a stratified Cox proportional hazards model, with the study group and stratification factors as covariates. Subgroup analysis was performed to detect the influences of various factors. Safety analysis was used to assess the toxic effects of the chemotherapy in patients presenting with grades 3 to 4 adverse events in each treatment group. Two-sided *P* < .05 indicated statistical significance.

## Results

### Patient Characteristics

**Figure 1.**
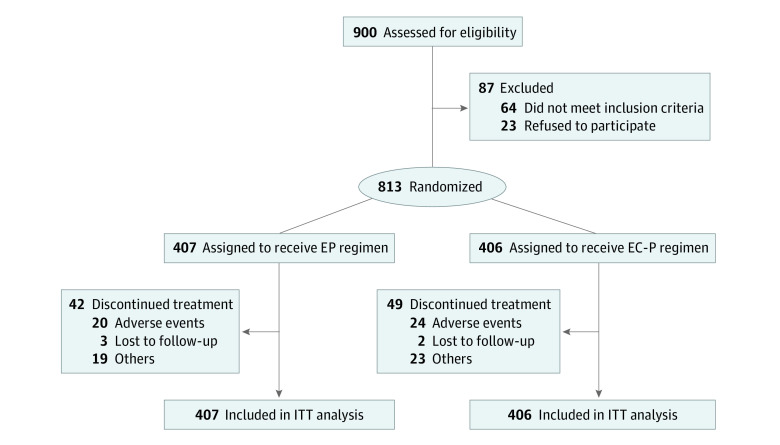
Study Flow Diagram EP indicates epirubicin plus paclitaxel; EC-P, epirubicin and cyclophosphamide followed by paclitaxel; ITT, intention to treat.

A total of 900 patients were registered between June 1, 2010, and June 30, 2016, and 813 eligible patients were randomized (1:1) to the EP group (n = 407) or the EC-P group (n = 406) after the surgical procedure as the ITT population (Figure 1). Patient characteristics and baseline clinicopathological variables were well balanced between the 2 groups (Table 1). For the whole population, the median age was 48 (IQR, 41-56) years. Among all patients, the Han population accounted for 792 (97.4%) and ethnic minority individuals accounted for 21 (2.6%), with no statistical difference between the 2 treatment groups. Luminal A tumors accounted for 267 patients (32.8%), and 243 patients (29.9%) had lymphovascular invasion.

**Table 1.** Baseline Patient and Tumor Characteristics^a^

Characteristic	Study group	
	EP (n = 407)	EC-P (n = 406)
Age, y		
Median (IQR) [range]	49 (42-56) [23-70]	48 (41-56) [24-77]
<55	287 (70.5)	294 (72.4)
≥55	120 (29.5)	112 (27.6)
Height, median (IQR) [range], cm	160 (156-164) [145-173]	160 (156-164) [141-176]
Weight, median (IQR) [range], kg	63 (57-70) [40-89]	63 (58-70) [40-105]
Tumor size, cm		
<2	210 (51.6)	212 (52.2)
2-5	175 (43.0)	178 (43.8)
>5	22 (5.4)	16 (3.9)
No. of nodes involved		
1-3	229 (56.3)	208 (51.2)
4-9	111 (27.3)	120 (29.6)
≥10	67 (16.5)	78 (19.2)
Histological grade		
1	26 (6.4)	13 (3.2)
2	277 (68.1)	276 (68.0)
3	104 (25.6)	117 (28.8)
TNM stage		
II	217 (53.3)	213 (52.5)
III	190 (46.7)	193 (47.5)
Lymphovascular invasion		
Yes	117 (28.7)	126 (31.0)
No	290 (71.3)	280 (69.0)
Luminal subtype		
A	143 (35.1)	124 (30.5)
B	264 (64.9)	282 (69.5)
Menopausal status (at diagnosis)		
Premenopausal	243 (59.7)	241 (59.4)
Postmenopausal	164 (40.3)	165 (40.6)
Ki67 level		
≤30%	303 (74.4)	284 (70.0)
>30%	65 (16.0)	77 (19.0)
Unknown	39 (9.6)	45 (11.1)
Type of surgery		
Modified radical mastectomy	341 (83.8)	347 (85.5)
Breast-conserving surgery	66 (16.2)	55 (13.5)
Endocrine therapy	394 (96.8)	393 (96.8)
Types of endocrine therapy		
Tamoxifen	153 (38.8)	147 (37.4)
Tamoxifen plus OFS	6 (1.5)	8 (2.0)
AI	130 (33.0)	122 (31.0)
AI plus OFS	94 (23.9)	106 (27.0)
Other^b^	11 (2.8)	10 (2.5)
Duration of endocrine therapy, y		
<2	20 (5.1)	24 (6.1)
2-5	88 (22.3)	115 (29.3)
>5	246 (62.4)	217 (55.2)
Unknown	40 (10.2)	37 (9.4)
Radiotherapy	257 (63.1)	258 (63.5)

Abbreviations: AI, aromatase inhibitor; EC-P, epirubicin and cyclophosphamide followed by paclitaxel; EP, epirubicin plus paclitaxel; OFS, ovarian function suppressor.

^a^ Unless otherwise indicated, data are presented as No. (%) of patients. Percentages have been rounded and may not total 100.

^b^ The regimens were changed during the endocrine therapy.

### Efficacy Analysis

**Figure 2.**
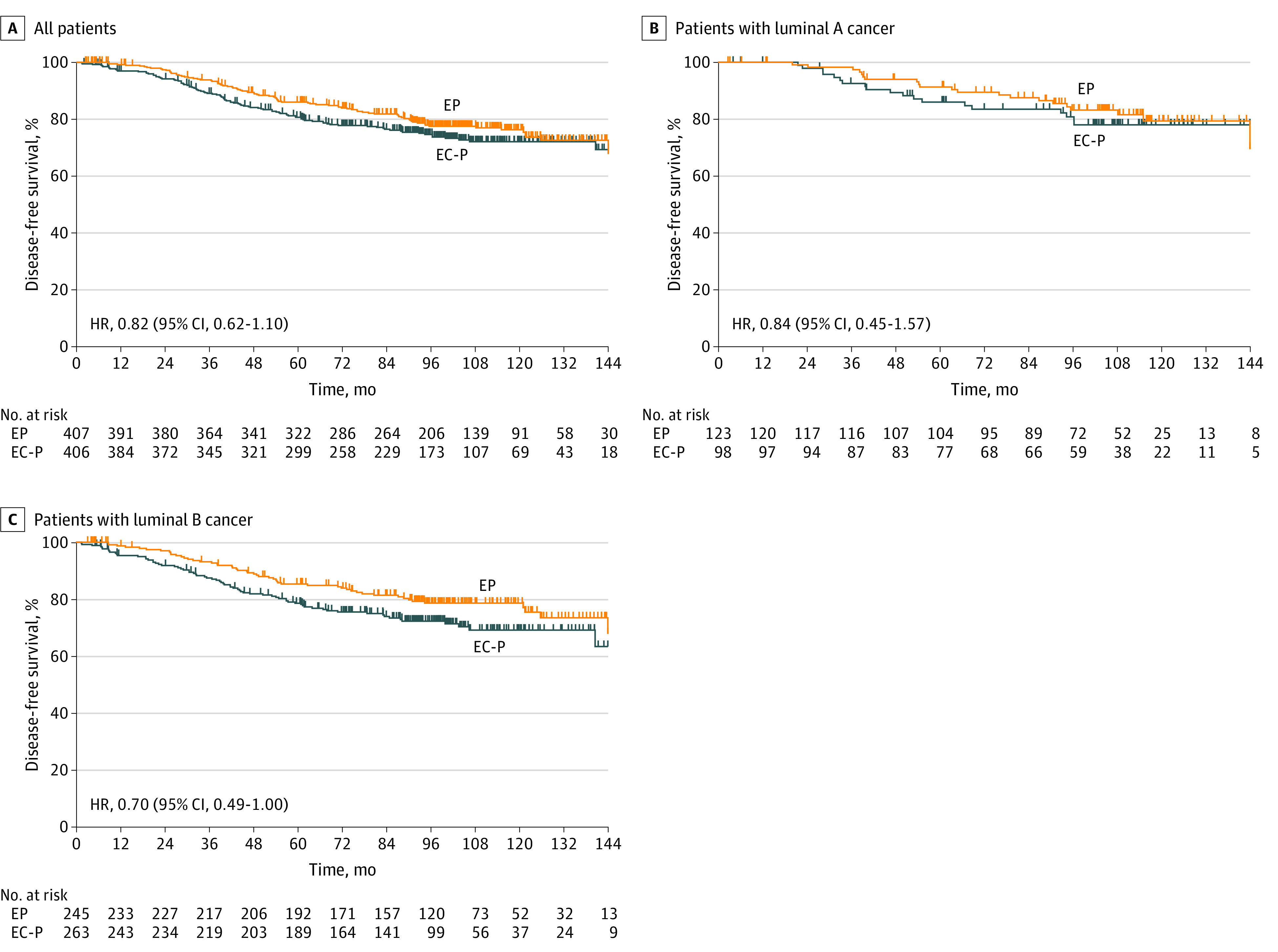
Kaplan-Meier Curves of Disease-Free Survival A, For all patients, those in the epirubicin plus paclitaxel (EP) group had 89 events; those in the epirubicin and cyclophosphamide followed by paclitaxel (EC-P) group, 100 events. B, For patients with luminal A tumors, patients in the EP group had 21 events; those in the EC-P group, 19 events. C, For patients with luminal B tumors, patients in the EP group had 51 events; those in the EC-P group, 71 events. HR indicates hazard ratio.

The median follow-up period was 93.6 (IQR, 60.9-114.1) months. There were 189 DFS events during the follow-up period, of which 89 were in the EP group and 100 in the EC-P group, respectively. The 5-year DFS for ITT population treated with EP or EC-P was 86.0% vs 80.6%, respectively (HR, 0.82 [95% CI, 0.62-1.10]; noninferior *P* = .001) (Figure 2). Therefore, it was conclusive that the EP group was noninferior to the EC-P group. The type of DFS events are illustrated in eTable 1 in Supplement 2. In the population with luminal A tumors, the DFS events for the EP and EC-P groups were 17.1% vs 19.4%, respectively (HR, 0.84 [95% CI, 0.45-1.57]). In the population with luminal B tumors, the DFS events for the EP and EC-P groups were 20.8% vs 27.0%, respectively (HR, 0.70 [95% CI, 0.49-1.00]).

**Figure 3.**
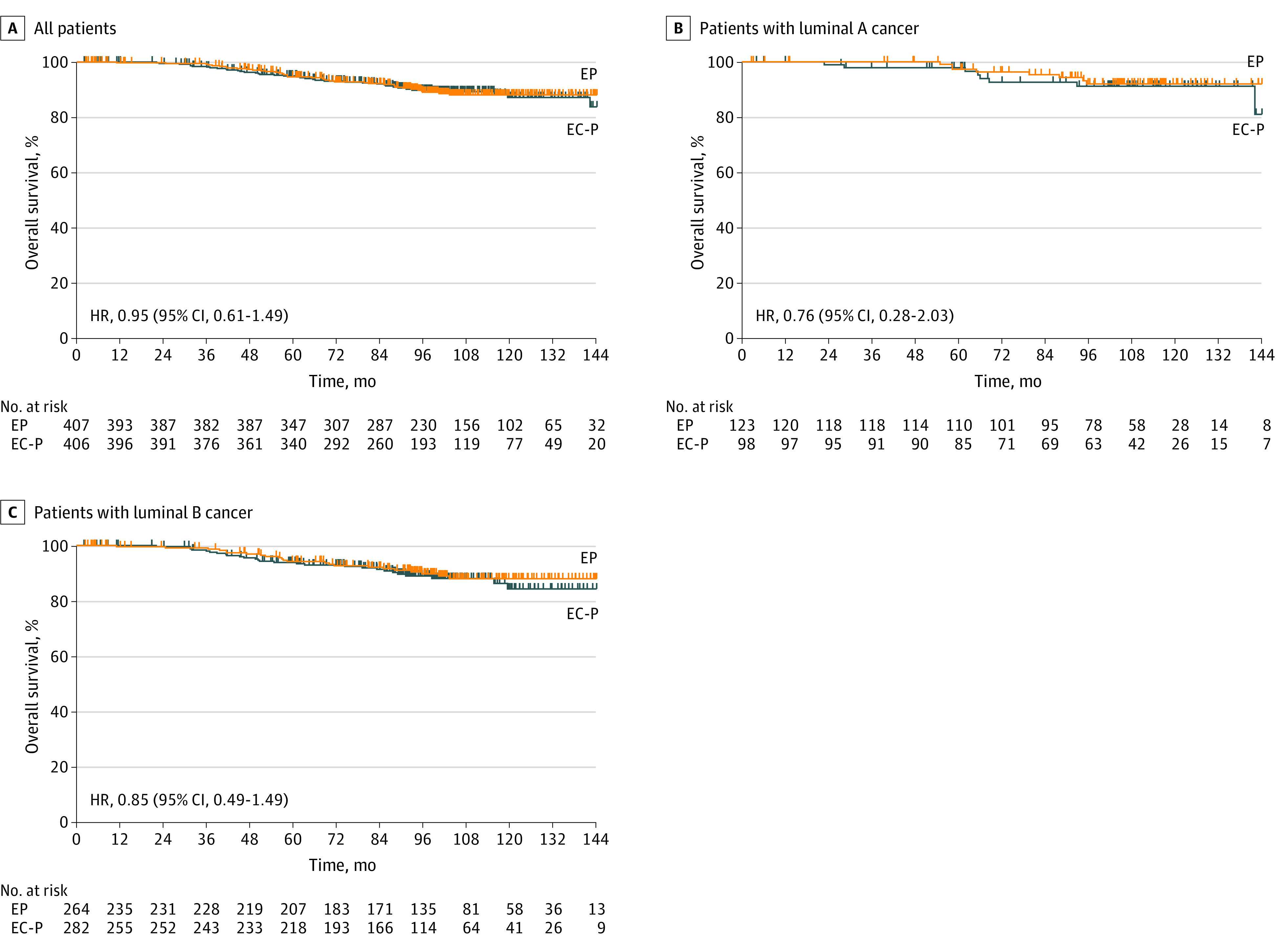
Kaplan-Meier Curves of Overall Survival A, For all patients, those in the epirubicin plus paclitaxel (EP) group had 39 events; those in the epirubicin and cyclophosphamide followed by paclitaxel (EC-P) group, 38 events. B, For patients with luminal A tumors, patients in the EP group had 8 events; those in the EC-P group, 8 events. C, For patients with luminal B tumors, patients in the EP group had 24 events; those in the EC-P group, 27 events. HR indicates hazard ratio.

There were 77 OS events during the follow-up period, of which 39 were in the EP group and 38 were in the EC-P group. The 5-year OS for the ITT population treated with EP or EC-P was 94.7% vs 95.0%, respectively (HR, 0.95 [95% CI, 0.61-1.49]) (Figure 3). In the population with luminal A tumors, the OS events for the EP and EC-P groups were 9.6% vs 9.4%, respectively (HR, 0.76 [95% CI, 0.28-2.03]). In the population with luminal B tumors, the OS events for the EP and EC-P groups were 6.5% vs 8.1%, respectively (HR, 0.85 [95% CI, 0.49-1.49]).

There were 161 DDFS events during the follow-up period, of which 73 were in the EP group and 88 were in the EC-P group. The 5-year DDFS for the ITT population treated with EP or EC-P was 88.4% vs 84.5%, respectively (HR, 0.77 [95% CI, 0.57-1.05]) (eFigure 1 in Supplement 2). In the population with luminal A tumors, the DDFS events for EP and EC-P were 13.8% vs 17.3%, respectively (HR, 0.76 [95% CI, 0.39-1.50]). In the population with luminal B tumors, the DDFS events for EP and EC-P were 17.6% vs 24.0%, respectively (HR, 0.68 [95% CI, 0.46-1.00]).

In addition, there were 217 DFS-s events during the follow-up period, of which 105 were in the EP group and 112 were in the EC-P group. The 5-year DFS-s for ITT population treated with EC-P or EP was 83.9% vs 79.1%, respectively (HR, 0.87 [95% CI, 0.66-1.14]) (eFigure 2 in Supplement 2). In the population with luminal A cancer, the DFS-s events for EP and EC-P were 21.1% vs 20.4%, respectively (HR, 1.05 [95% CI, 0.64-1.58]). In the population with luminal B cancer, the DFS-s events for EP and EC-P were 25.3% vs 30.8%, respectively (HR, 0.81 [95% CI, 0.59-1.11]).

Subgroup analysis was stratified by age, menopausal status, subtype, tumor grade, Ki67 level, lymphovascular invasion, endocrine therapy, and radiotherapy (eTable 2 in Supplement 2). Subgroup analysis showed similar treatment effects between the EP and EC-P groups by age, subtype, tumor grade, Ki67 level, lymphovascular invasion, and endocrine therapy, but indicated differential effects in postmenopausal patients (DFS events: 20.1% vs 30.3%; HR, 0.59 [95% CI: 0.38-0.91) and in patients without radiotherapy (DFS events: 14.0% vs 23.6%; HR, 0.55 [95% CI, 0.32-0.93]).

### Safety Analysis

Adverse events were recorded for patients who received at least 1 dose of allocated chemotherapy (Table 2). No treatment-related death occurred during the treatment. Compared with the EC-P group, patients in the EP group had more frequent toxic effect events, including any grade of leukopenia, neutropenia, anemia, thrombocytopenia, gastrointestinal tract toxic effects, neurotoxic effects, hepatotoxic effects, cardiotoxic effects, alopecia, and fatigue. Grades 3 to 4 leukopenia and neutropenia also occurred more frequently in the EP group. In premenopausal cases, the percentage of those achieving chemical menopause was higher in the EC-P group (eTable 3 in Supplement 2).

**Table 2.** Safety Analysis^a^

Adverse effect	Study group					
	EP (n = 407)			EC-P (n = 406)		
	Any grade	Grade 3	Grade 4	Any grade	Grade 3	Grade 4
Leukopenia	301 (74.0)	153 (37.6)	62 (15.2)	281 (69.2)	134 (33.0)	38 (9.4)
Neutropenia	295 (72.5)	59 (14.5)	196 (48.2)	268 (66.0)	92 (22.7)	131 (32.3)
Anemia	194 (47.7)	13 (3.2)	0	132 (32.5)	7 (1.7)	0
Thrombocytopenia	51 (12.5)	4 (1.0)	0	45 (11.1)	7 (1.7)	2 (0.5)
Gastrointestinal tract toxic effects	337 (82.8)	20 (4.9)	0	281 (69.2)	23 (5.7)	0
Neurotoxic effects	159 (39.1)	6 (1.5)	0	146 (36.0)	14 (3.4)	0
Hepatotoxic effects	129 (31.7)	5 (1.2)	1 (0.2)	111 (27.3)	7 (1.7)	0
Cardiotoxic effects	107 (26.3)	0	0	98 (24.1)	1 (0.2)	0
Alopecia	246 (60.4)	67 (16.5)	1 (0.2)	186 (45.8)	47 (11.6)	0
Fatigue	65 (16.0)	0	0	60 (14.8)	1 (0.2)	0
Hyperpigmentation	7 (1.7)	0	0	11 (2.7)	0	0

Abbreviations: EC-P, epirubicin and cyclophosphamide followed by paclitaxel; EP, epirubicin plus paclitaxel.

^a^ Data are presented as No. (%) of patients.

## Discussion

This trial was designed to determine whether the EP regimen is noninferior to the standard EC-P regimen in both efficacy and safety for *ERBB2*-negative breast cancer. The results demonstrate that EP regimen reaches the noninferior standard to the EC-P regimen mainly in operable hormone receptor–positive, *ERBB2*-negative, node-positive breast cancer. To the best of our knowledge, this is the first phase 3 clinical trial to compare the long-term outcomes of 2 adjuvant chemotherapy regimens, specifically in Asian patients with operable breast cancer.

Cyclophosphamide is an important component of most adjuvant chemotherapy regimens in breast cancer.^15,16^ However, research has repeatedly demonstrated that it also contributes substantially to the risk of gonadotoxic effects, especially in premenopausal women.^17,18^ Previous meta-analysis^19^ compared the efficacy and safety of anthracycline plus taxane–based neoadjuvant chemotherapy in patients with breast cancer. The results showed that the anthracycline plus taxane–based neoadjuvant chemotherapy regimen with or without cyclophosphamide had similar clinical outcomes in patients with breast cancer, and the addition of cyclophosphamide could increase the risks of thrombocytopenia, sensory and/or motor neuropathy, and nausea and vomiting. Therefore, the feasibility of cyclophosphamide-free regimens has been investigated in breast cancer. The SPECTRUM (Substitution of Paclitaxel for Cyclophosphamide on Survival Outcomes and Resumption of Menses in Young Women with ER [Estrogen Receptor]–Positive Breast Cancer) trial^20^ was designed to compare the survival outcomes of standard adjuvant epirubicin plus cyclophosphamide followed by weekly paclitaxel (EC-wP) and epirubicin plus paclitaxel followed by weekly paclitaxel (EP-wP) in young women with breast cancer. In that trial, at a median follow-up of 62 months, the 5-year DFS was 78.3% (95% CI, 72.2%-83.3%) in the EC-wP group and 84.7% (95% CI, 79.3%-88.8%) in the EP-wP group (*P* = .07). Additionally, the rate of menstrual resumption at 12 months after chemotherapy was 48.3% (95% CI, 42.2%-54.3%) in the EC-wP group and 63.1% (95% CI, 57.2%-68.9%) in the EP-wP group, with an absolute difference of 14.8% (95% CI, 6.37%-23.2%; *P* < .001). The patient-reported questionnaires indicated that pregnancy might occur in fewer women in the EC-wP group than in the EP-wP group. Different from our study, the SPECTRUM trial focused on women who were younger than 40 years.

The Cancer and Leukemia Group B (CALGB) 9741 trial^7^ compared the adjuvant chemotherapy regimen sequential doxorubicin, paclitaxel, and cyclophosphamide with concurrent doxorubicin and cyclophosphamide followed by paclitaxel in women with axillary node-positive breast cancer. In that trial, the dose-dense treatment improved DFS (risk ratio, 0.74; *P* = .01) and OS (risk ratio, 0.69; *P* = .01). Four-year DFS was 82% for the dose-dense regimens and 75% for the others (*P* = .01). In our study, the 5-year DFS of the group receiving the 6-cycle EP regimen reached 86%, which was higher than the EC-P regimen in *ERBB2*-negative, node-positive breast cancer.

It seems that the differences in DFS and DDFS between the 2 groups are not reflected at all in the OS curve. Several reasons may account for it. First, OS is a parameter with all-cause death, which can be influenced by various factors. Although the relapse rate seems a little different in the 2 groups, further management is also crucial to affect OS. Second, DFS and DDFS are better in patients with luminal B breast cancer. With higher levels of Ki67, the patients with the luminal B subtype may have higher risk of relapse; therefore, those patients may benefit more from chemotherapy like the EP regimen.

In the present study, subgroup analysis showed that the EP group had a better DFS than the EC-P group among postmenopausal women and patients without radiotherapy. These results indicate that the EP regimen may be more suitable for postmenopausal women and patients who are unable to receive radiotherapy. However, this discrepancy may have resulted from the higher dose of epirubicin and paclitaxel used in the EP regimen. Combined with the former studies,^7,20^ our data suggest the feasibility of cyclophosphamide-free regimens in *ERBB2*-negative breast cancer.

In addition, whether a sequential or concurrent regimen of anthracyclines and taxanes is superior for breast cancer is controversial. A recent meta-analysis detected that the sequential regimen of anthracyclines and taxanes for patients with operable breast cancer did not show a significant benefit in DFS or OS over the concurrent regimen.^21^ However, the sequential regimen demonstrated a better DFS than the concurrent regimen for patients with node-positive cancer. Further subgroup analysis also revealed that for patients with node-positive cancer who were given doxorubicin and taxanes, more cycles (6 cycles) of the concurrent regimen might rescue the efficacy for fewer cycles (4 cycles).

In this study, the patients in the EP group had more frequent toxic effect events than those in the EC-P group, including any grade of leukopenia, neutropenia, anemia, thrombocytopenia, gastrointestinal tract toxic effects, neurotoxic effects, hepatotoxic effects, cardiotoxic effects, alopecia, and fatigue. Grades 3 and 4 leukopenia and neutropenia also occurred more frequently in the EP group, likely because epirubicin and paclitaxel were administered concurrently during the same treatment period. Although the total dose of anthracyclines was increased, grades 3 and 4 cardiotoxic effects were not observed, and cardiotoxic effects were within a manageable range. Severe myelosuppression can be prevented by the administration of granulocyte colony-stimulating factor. Other severe adverse effects were also treated and managed, and no treatment-related death occurred in both groups during the treatment.

In addition to the cyclophosphamide-free regimen, other studies^22^ have also revealed the anthracycline-free regimen in women with breast cancer. Yu et al^22^ evaluated the noninferiority of an anthracycline-free or short-term regimen to the standard anthracycline-based regimen for patients with operable *ERBB2*-negative breast cancer. The patients were randomized to 6 cycles of docetaxel and cyclophosphamide, or epirubicin and cyclophosphamide for 4 cycles followed by paclitaxel for 12 weeks (EC-P). Through a median follow-up of 5.5 (IQR, 3.5-6.7) years, HR for docetaxel and cyclophosphamide vs EC-P was 1.05 (90% CI, 0.79-1.39; 5-year DFS, 85.0% vs 85.9%, respectively; noninferior *P* = .05), which showed noninferiority of the docetaxel and cyclophosphamide regimen compared with the EC-P regimen.

### Strengths and Limitations

Our study revealed the feasibility of a cyclophosphamide-free regimen as adjuvant chemotherapy in operable *ERBB2*-negative breast cancer. However, some limitations should be noted when interpreting the main results. First, this open-label study was performed in a single center, which might limit its application. Second, the clinical trials about adjuvant treatment often require a large sample size and long-term observation to draw reliable conclusions. Therefore, the sample size in this study should be larger. Third, we used 3 cycles of EC-P as the standard regimen rather than the dose-dense regimen that has been recommended by the latest version of the National Comprehensive Cancer Network Clinical Practice Guidelines in Oncology.^23^ This is because the dose-dense EC-P regimen has been widely applied as the standard regimen since 2017 in China, but all included patients in this study were enrolled between 2010 to 2016. Fourth, though subgroup analysis shows various results, the subgroup analysis is underpowered due to the small number of cases. Fifth, we did not record and analyze the impact of the cyclophosphamide-free regimen on the gonadotoxic effects in young women. Sixth, subpopulation treatment effect pattern plot analysis was not performed to get a deeper insight into the interpretation of the data. Finally, since this study was designed more than 10 years ago, some innovative therapies were not applied. In the era of genetic testing, information on gene predisposition might provide new insights. Adjuvant chemotherapy guided by a 21-gene expression assay has been proved in breast cancer.^24^ We did not analyze the status of some well-known gene mutations, such as *BRCA1/2* and *RAD51C/D*.^25,26^ This important information may also affect the results of our study.

## Conclusions

In this randomized clinical trial, the long-term efficacy of the EP regimen was noninferior to the EC-P regimen. Our findings also show that the EP regimen was an effective adjuvant chemotherapy regimen for women with *ERBB2*-negative breast cancer. However, it is essential for clinicians to pay more attention to adverse effects of EP regimen to promote medication safety.

## Notes

Supplement 1.
Trial Protocol

Supplement 2. **eFigure 1.** Kaplan-Meier Curves of Distant Disease-Free Survival (DDFS) in All Patients (A), Patients With Luminal A Cancer (B), and Patients With Luminal B Cancer (C) **eFigure 2.** Kaplan-Meier Curves of Disease-Free Survival Including Second Nonbreast Invasive Cancer (DFS-s) in All Patients (A), Patients With Luminal A Cancer (B), and Patients With Luminal B Cancer (C) **eTable 1.** The Comparison of Disease-Free Survival Between the 2 Groups **eTable 2.** Subgroup Analysis **eTable 3.** The Comparison of Chemical Menopause Events Between the 2 Groups

Supplement 3.
Data Sharing Statement
