# Supplementary material for: Effect of Epirubicin Plus Paclitaxel vs Epirubicin and Cyclophosphamide Followed by Paclitaxel on Disease-Free Survival Among Patients With Operable ERBB2-Negative and Lymph Node–Positive Breast Cancer: A Randomized Clinical Trial
Source: JAMA Netw Open. 2023 Feb 24;6(2):e230122. doi: 10.1001/jamanetworkopen.2023.0122 (PMC9958529; doi:10.1001/jamanetworkopen.2023.0122)
Supplement: Supplement 1. — Trial Protocol [file jamanetwopen-e230122-s001.pdf]

Supplement 1:Trial Protocol

List of Contents:

1. Inclusion Criteria
2. Exclusion Criteria
3. Dose and schedule
4. Dose modifications
5. Endpoints
6. Sample Calculation
7. Pathologic evaluation method
8. Supplementary References

1. Inclusion Criteria:

- Written informed consent
- Female patients, age at diagnosis＞18 years
- Karnofsky≥70
- Histological confirmed unilateral primary invasive carcinoma of the breast with node-positive (pN +) tumors
- Human epidermal growth factor receptor 2 (HER2) negative judged by two pathologists according to updated guidelines of the American Society of Clinical Oncology/College of American Pathologists (ASCO/CAP) [1,2]
- Hormone receptor positive
- No evidence for distant metastasis (M0) after conventional staging
- The patient must be accessible for treatment and follow-up
- Negative pregnancy test (urine or serum) within 7 days prior to randomization in premenopausal patients
- Adequate organ and bone marrow function as evidenced by
  1. Leucocytes ≥4 x 10^9/L
  2. Platelets ≥100 x 10^9/L
  3. Hemoglobin ≥9 g/dL
  4. Total bilirubin ≤1.5 UNL
  5. Aspartate transaminase (AST) and alanine transaminase (ALT) ≤2.5 UNL
  6. Creatinine <175 mmol/L (2 mg/dL)
- Left ventricular ejection fraction (LVEF) >50%

2. Exclusion Criteria

- Pregnant, or breast feeding, or women of childbearing age who cannot practice effective contraceptives
- Has previous history of additional malignancy, with the exception of adequately treated basal cell carcinoma and cervical carcinoma in situ
- Has received neoadjuvant therapy (include chemotherapy, targeted therapy, radiotherapy or endocrine therapy)
- Has metastatic (Stage 4) breast cancer
- Has any >T4 lesion (UICC1987) (with skin involvement, mass adhesion and fixation, and inflammatory breast cancer)
- Patients participating in other clinical trials at the same time
- Has severe organ dysfunction (cardiopulmonary liver and kidney) insufficiency, LVEF <50% (cardiac ultrasound); severe cardio cerebral vascular disease within the 6 months previous of randomization (such as unstable angina, chronic heart failure, uncontrolled hypertension with blood pressure >150/90mmHg, myocardial infarction, or cerebral blood vessel); diabetic patients with poor blood glucose control; patients with severe hypertension
- Has known allergy to taxanes and excipients
- Has severe or uncontrolled infection
- Inability to comply with study and follow-up procedure
- Any other finding giving reasonable suspicion of a disease or condition that contraindicates the treatment

3. Dose and schedule:

Arm A: epirubicin (75 mg/m2) and paclitaxel (175 mg/m2) every 3 weeks for 6 cycles (EP)

Arm B: epirubicin 90 mg/m2 and cyclophosphamide 600 mg/m2 every 3 weeks for 4 cycles followed by paclitaxel 175 mg/m2 every 3 weeks for 4 cycles (EC-P)

Upon completion of treatment, patients underwent follow up surveillance and were scheduled to be seen every 3 months for the first two years and every 6 months after that for 10 years.

Chemotherapy was administered before radiation therapy if radiation was indicated. Radiotherapy was completed by patients who received breast conservation or with ≥4 involved axillary lymph nodes or those with 1-3 involved axillary lymph nodes along with other high-risk factors. On completion of chemotherapy and/or radiotherapy, endocrinotherapy (the regimen was decided by the physicians) was administered to patients for 5 years.

4. Dose modifications:

4.1 Treatment interrupted or aborted

In the following scenario, patients should discontinue their allocated treatment:

- Medical conditions that are harmful to patients' health judged by investigators
- Unacceptable toxicity, for example: more than twice dose delay and/or dose reduction due to hematologic toxicity reduction twice; grade 3 or 4 non-hematologic toxicity occurs for the third time
- Patients still cannot receive the designated treatment for 42 days after the last administration of previous cycle
- Patient requirements
- Relapse of the disease
- Poor compliance

If a patient withdraws from the study treatment, every effort should be made to keep up-to-date on the annual survival status, to get the latest survival status for at least 5 years after the enrollment, and to make effective efforts to determine the reason why the patient cannot follow up or withdraw from the trial. If the patient stops the treatment or follow-up, detailed cause must be recorded and the patient who withdrew from the trial cannot be replaced.

4.2 Dose reduction

Dose reduction is allowed according to the severest level of overall toxicity. Drug reduction due to hematological or non-hematologic toxic reactions is permanent.

- Hematological toxicity

  Dose reductions caused by hematological toxicity should be based on the lowest levels of neutrophils and platelet counts tested after the previous cycle, following the table below:

Table 1. Dose adjustment during the cycle based on the minimum count of neutrophils caused by the previous cycle**Error! Bookmark not defined.**

**Neutrophils ($\times 10^9$/L)**	**Dose of paclitaxel**	**Dose of epirubicin**	**Dose of cyclophosphamide**
> 1.5	100%	100%	100%
1.0-1.49	75%	75%	75%
< 1.0	Dose delay	Dose delay	Dose delay

- Non-hematological toxicity

Dose reduction due to non-hematologic toxicity will be implemented following the tables below:

Table 2. Dose adjustment during the cycle based on the non-hematological toxicity

**Toxicity**	**Grade**	**Dose of paclitaxel**	**Dose of epirubicin /cyclophosphamide**
**Liver**	0-2	100%	100%
	3	50%	50%
	4	Dose delay	Dose delay
**Myalgia or peripheral neuropathy**	0-1	100%	100%
	2	50%	50%
	3/4	Dose delay	Dose delay
**Mucositis**	0-2	100%	100%
	3	50%	50%
	4	Dose delay	Dose delay
**Bradycardia with symptoms**	Any	Dose delay	Dose delay
**Other toxicity (except nausea, vomiting and hair loss)**	0-2	100%	100%
	3	50%	50%
	4	Dose delay	Dose delay

5. Endpoints

The primary endpoint was DFS, defined as the time from randomization to occurrence of a new event including local recurrence, regional relapse, distant metastasis, or death from any cause (excluding second non-breast invasion). Patients alive without any predefined event were censored at the time of the last follow-up. Secondary endpoints included: (1) OS, defined as the time from randomization to death from any cause; (2) DFS-s, defined as the time from randomization to occurrence of a new event including local recurrence, regional relapse, distant metastasis, and second non-breast invasive cancer; and (3) safety, which was assessed throughout the study treatment according to the Common Terminology Criteria for Adverse Events (CTCAE), version 4.0.

6. Sample Calculation:

This trial was designed to evaluate the non-inferiority of EP versus EC-P. The test was designed with 80% power at the one-sided alpha of 0.05. The trial assumed a 5-year DFS of 83% for EC-P. [3,4] Non-inferiority was defined as the 5-year DFS of EP being not worse than an absolute value of 5% below EC-P, with a limiting hazard ratio (HR) of 1.30. Under these assumptions, the sample size was approximate 800 patients, with a ratio of 1:1 in each group. HRs were obtained using the Cox proportional hazards model. Non-inferior P values were calculated according to previous study. [5]

7. Pathologic evaluation method:

Primary surgically removed tumour tissues were sent to the Department of Pathology in National Cancer Center/National Clinical Research Center for Cancer/Cancer Hospital, Chinese Academy of Medical Sciences and Peking Union Medical College for slide review, immunohistochemistry (IHC) staining, and fluorescence *in situ* hybridization (FISH) analysis. Two experienced breast pathologists assessed histology and central grade and were both blinded to the clinical data and to Ki67 expression. Slides were stained for estrogen receptor (ER) (rabbit [SP1]; Neomarkers, Fremont, CA), progesterone receptor (PR) (mouse monoclonal PgR636; DAKO, Glostrup, Denmark), and Ki67 (clone 30-9 rabbit monoclonal; Ventana, Tucson, AZ) using standard protocols. Tumours were classified as ER or PR positive if IHC was present in ≥1% of tumour nuclei. Ki67 was evaluated in at least 100 tumour cells within the high-density area semi-quantitatively (in 5% increments) and quantitatively (in 1% increments). Additionally, the patients with HER2 expression status (IHC, score =2) were subjected to fluorescence in situ hybridization (FISH) screening for HER2 gene amplification. The HER2 negative subgroup was defined as FISH negative with IHC score<3 or IHC score<2.

8. Supplementary References:

1. Wolff AC, Hammond ME, Schwartz JN, et al. American Society of Clinical Oncology/College of American Pathologists guideline recommendations for human epidermal growth factor receptor 2 testing in breast cancer. *J Clin Oncol.* 2007;25(1):118-145.
2. Wolff AC, Hammond ME, Hicks DG, et al. Recommendations for human epidermal growth factor receptor 2 testing in breast cancer: American Society of Clinical Oncology/College of American Pathologists clinical practice guideline update. *J Clin Oncol.* 2013;31(31):3997-4013.
3. Swain SM, Tang G, Geyer CE Jr, et al. Definitive results of a phase III adjuvant trial comparing three chemotherapy regimens in women with operable, node-positive breast cancer: the NSABP B-38 trial. J Clin Oncol 2013;31:3197-204.
4. Swain SM, Jeong JH, Geyer CE Jr, et al. Longer therapy, iatrogenic amenorrhea, and survival in early breast cancer. N Engl J Med 2010;362:2053-65.
5. Tunes da Silva G, Logan BR, Klein JP. Methods for equivalence and noninferiority testing. Biol Blood Marrow Transplant 2009;15:120-7.
