# Supplementary material for: Effect of Epirubicin Plus Paclitaxel vs Epirubicin and Cyclophosphamide Followed by Paclitaxel on Disease-Free Survival Among Patients With Operable ERBB2-Negative and Lymph Node–Positive Breast Cancer: A Randomized Clinical Trial
Source: JAMA Netw Open. 2023 Feb 24;6(2):e230122. doi: 10.1001/jamanetworkopen.2023.0122 (PMC9958529; doi:10.1001/jamanetworkopen.2023.0122)
Supplement: Supplement 2. — eFigure 1. Kaplan-Meier Curves of Distant Disease-Free Survival (DDFS) in All Patients (A), Patients With Luminal A Cancer (B), and Patients With Luminal B Cancer (C) eFigure 2. Kaplan-Meier Curves of Disease-Free Survival Including Second Nonbreast Invasive Cancer (DFS-s) in All Patients (A), Patients With Luminal A Cancer (B), and Patients With Luminal B Cancer (C) eTable 1. The Comparison of Disease-Free Survival Between the 2 Groups eTable 2. Subgroup Analysis eTable 3. The Comparison of Chemical Menopause Events Between the 2 Groups [file jamanetwopen-e230122-s002.pdf]

Supplementary Online Content

Yuan P, Kang Y, Ma F, et al. Effect of epirubicin plus paclitaxel vs epirubicin and cyclophosphamide followed by paclitaxel on disease-free survival among patients with operable *ERBB2*-negative and lymph node–positive breast cancer: a randomized clinical trial. *JAMA Netw Open*. 2023;6(2):e230122. doi:10.1001/jamanetworkopen.2023.0122

eFigure 1. Kaplan-Meier Curves of Distant Disease-Free Survival (DDFS) in All Patients (A), Patients With Luminal A Cancer (B), and Patients With Luminal B Cancer (C)
eFigure 2. Kaplan-Meier Curves of Disease-Free Survival Including Second Nonbreast Invasive Cancer (DFS-s) in All Patients (A), Patients With Luminal A Cancer (B), and Patients With Luminal B Cancer (C)
eTable 1. The Comparison of Disease-Free Survival Between the 2 Groups
eTable 2. Subgroup Analysis
eTable 3. The Comparison of Chemical Menopause Events Between the 2 Groups

This supplementary material has been provided by the authors to give readers additional information about their work.

© 2023 Yuan P et al. *JAMA Network Open*.

eFigure 1. Kaplan-Meier Curves of Distant Disease-Free Survival (DDFS) in All Patients (A), Patients With Luminal A Cancer (B), and Patients With Luminal B Cancer (C)

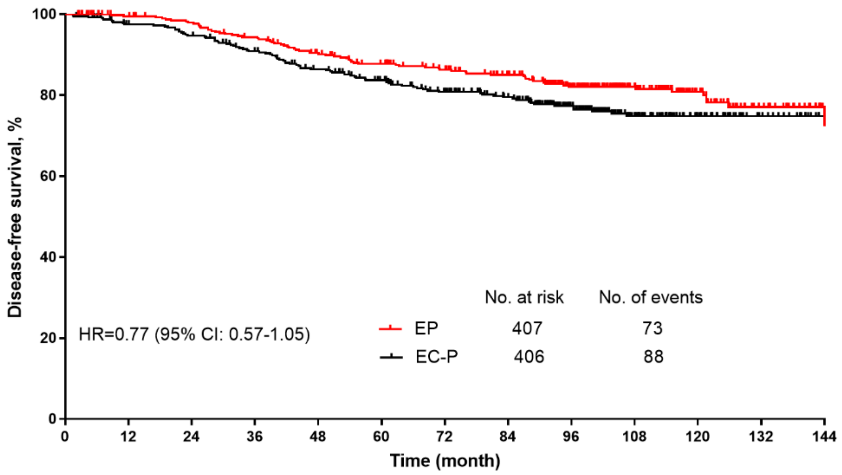

No. at risk (No. of events)

EP	407	392	381	365	344	326	292	273	218	149	97	63	32
EC-P	406	386	374	352	329	308	266	238	179	110	72	45	19

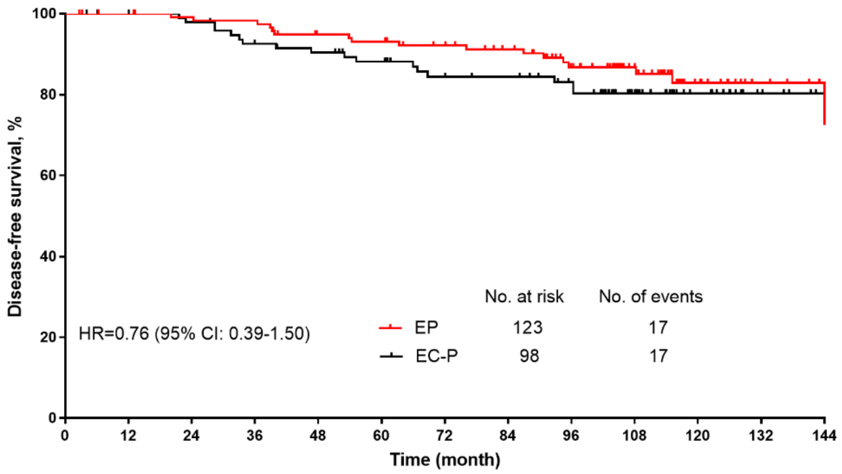

No. at risk (No. of events)

EP	123	120	117	116	108	106	98	92	75	55	26	14	8
EC-P	98	97	94	87	84	79	69	67	61	40	24	13	6

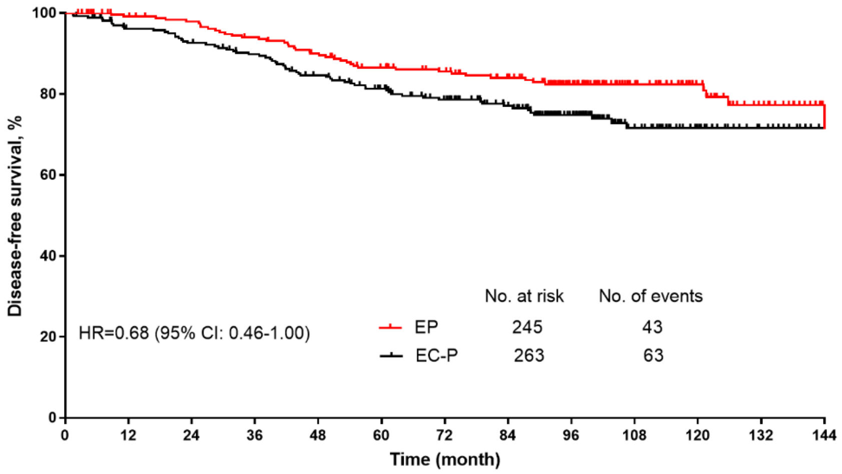

No. at risk (No. of events)

EP	245	234	228	218	207	193	174	161	126	78	55	35	13
EC-P	263	245	236	225	209	193	169	146	102	57	38	24	9

© 2023 Yuan P et al. *JAMA Network Open*.

eFigure 2. Kaplan-Meier Curves of Disease-Free Survival Including Second Nonbreast Invasive Cancer (DFS-s) in All Patients (A), Patients With Luminal A Cancer (B), and Patients With Luminal B Cancer (C)

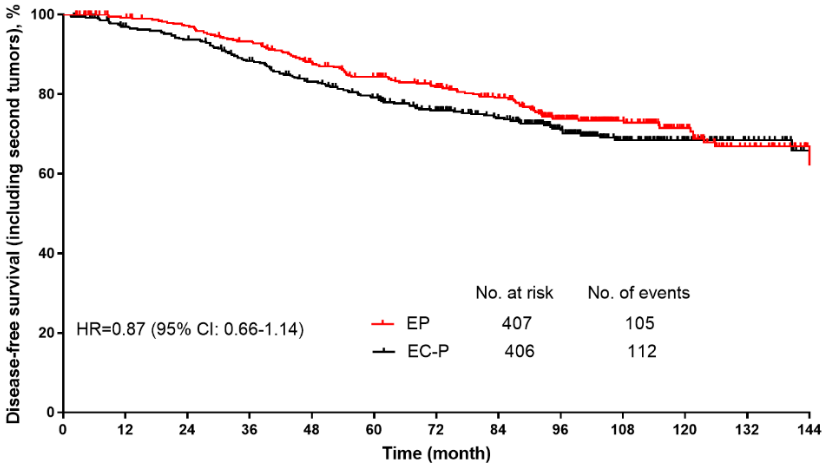

No. at risk (No. of events)

EP	407	391	379	362	336	316	278	254	198	132	87	56	28
EC-P	406	384	370	343	318	295	253	222	167	105	68	42	18

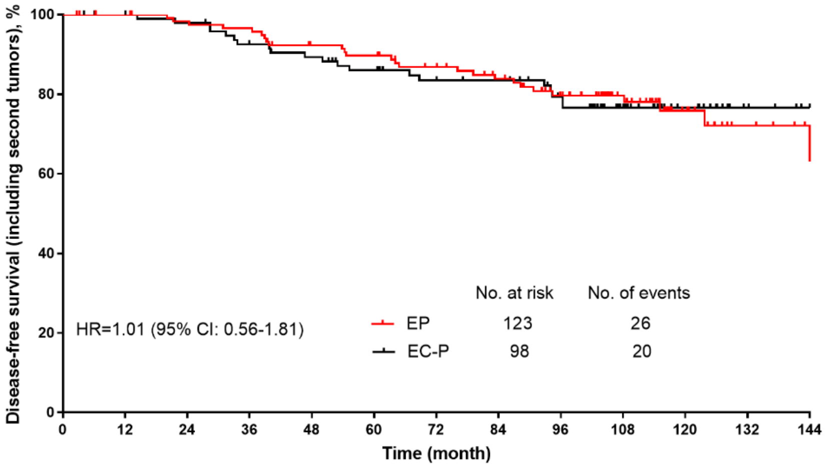

No. at risk (No. of events)

EP	123	140	136	129	118	115	101	94	78	58	31	19	13
EC-P	98	123	120	110	103	95	79	75	65	45	28	17	8

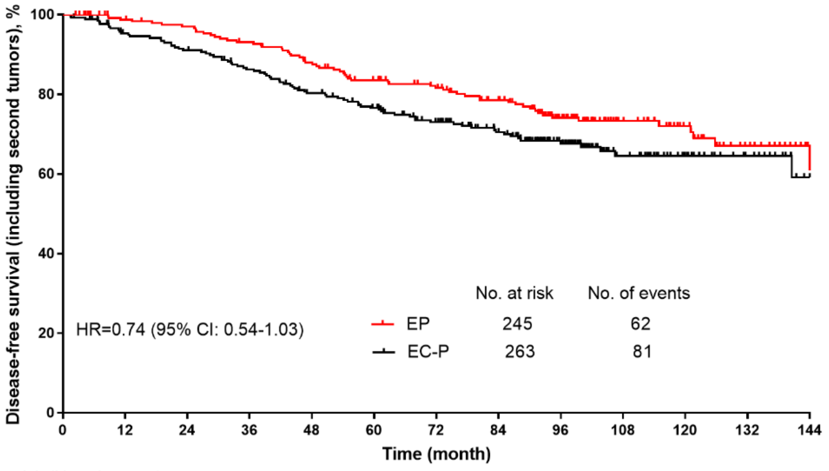

No. at risk (No. of events)

EP	245	233	227	217	203	188	166	151	113	67	49	30	11
EC-P	263	243	232	217	200	186	160	135	94	55	37	24	9

© 2023 Yuan P et al. *JAMA Network Open*.

eTable 1. The Comparison of Disease-Free Survival Between the 2 Groups

	EP (n=407)	EC-P (n=406)
Patients with an event	89 (21.9)	100 (24.6)
Breast cancer relapse	82 (20.1)	95 (23.4)
Local recurrence	8 (2.0)	10 (2.5)
Regional recurrence	17 (4.2)	29 (7.1)
Distant recurrence	64 (15.7)	74 (18.2)
Bone	34 (8.4)	47 (11.6)
Lung	22 (5.4)	27 (6.7)
Liver	20 (4.9)	26 (6.4)
Brain	2 (0.5)	2 (0.5)
Other	6 (1.5)	5 (1.2)
Death	7 (1.7)	5 (1.2)

© 2023 Yuan P et al. *JAMA Network Open*.

eTable 2. Subgroup Analysis

	DFS							OS					
	EP		EC-P		HR (95% CI)	*P* value		EP		EC-P		HR (95% CI)	*P* value
	No.	Events	No.	Events				No.	Events	No.	Events		
Age													
<55 years	287	65	294	68	0.93 (0.66-1.30)	0.652		287	24	294	28	1.15 (0.66-1.96)	0.629
≥55 years	120	24	112	32	0.60 (0.35-1.02)	0.059		120	11	112	14	0.62 (0.28-1.37)	0.235
Menopausal status													
Premenopausal	243	56	241	50	1.05 (0.72-1.54)	0.775		243	24	241	17	1.32 (0.71-2.44)	0.373
Postmenopausal	164	33	165	50	0.59 (0.38-0.91)	0.017		164	15	165	21	0.66 (0.34-1.27)	0.214
TNM stage													
Stage2	217	31	213	30	0.93 (0.56-1.54)	0.774		217	10	213	7	1.27 (0.49-3.33)	0.622
Stage3	190	58	193	70	0.78 (0.55-1.10)	0.155		190	29	193	31	0.91 (0.55-1.52)	0.719
Ki67													
≤30%	303	57	284	67	0.72 (0.51-1.03)	0.076		303	24	284	23	0.90 (0.51-1.59)	0.715
>30%	65	15	77	23	0.76 (0.40-1.45)	0.408		65	7	77	12	0.72 (0.29-1.79)	0.490
Lymphovascular invasion													
Yes	117	32	126	33	1.03 (0.63-1.67)	0.902		117	17	126	16	1.14 (0.57-2.22)	0.725
No	290	57	280	67	0.74 (0.52-1.05)	0.099		290	22	280	22	0.89 (0.49-1.61)	0.700
Endocrinotherapy													
Yes	394	86	393	97	0.82 (0.61-1.09)	0.172		394	38	393	37	0.95 (0.61-1.49)	0.835
No	13	3	13	3	1.10 (0.22-5.56)	0.908		13	1	13	1	1.09 (0.07-16.67)	0.953
Radiotherapy													
Yes	257	68	258	65	0.98 (0.69-1.37)	0.894		257	30	258	26	1.06 (0.63-1.82)	0.808
No	150	21	148	35	0.55 (0.32-0.93)	0.024		150	9	148	12	0.72 (0.31-1.69)	0.462

DFS, disease-free survival; OS, overall survival; HR, hazard ratio; CI, confidence interval.

© 2023 Yuan P et al. *JAMA Network Open*.

eTable 3. The Comparison of Chemical Menopause Events Between the 2 Groups

	EP (n=135) [a]	EC-P (n=114) [a]
CIA	61 (45.2)	59 (51.8)

[a] Those who were postmenopausal, or with irregular menstruation, or received ovarian function inhibitors were excluded from the analysis. CIA, chemotherapy-induced amenorrhea, which was defined as amenorrhea for more than 12 months during the chemotherapy or within 1 year after the chemotherapy.

© 2023 Yuan P et al. *JAMA Network Open*.
