# Supplementary material for: Effect of Epirubicin Plus Paclitaxel vs Epirubicin and Cyclophosphamide Followed by Paclitaxel on Disease-Free Survival Among Patients With Operable ERBB2-Negative and Lymph Node–Positive Breast Cancer: A Randomized Clinical Trial
Source: JAMA Netw Open. 2023 Feb 24;6(2):e230122. doi: 10.1001/jamanetworkopen.2023.0122 (PMC9958529; doi:10.1001/jamanetworkopen.2023.0122)
Supplement: Supplement 3. — Data Sharing Statement [file jamanetwopen-e230122-s003.pdf]

Data Sharing Statement

Yuan. Effect of Epirubicin Plus Paclitaxel vs Epirubicin and Cyclophosphamide Followed by Paclitaxel on Disease-Free Survival Among Patients With Operable ERBB2-Negative and Lymph Node-Positive Breast Cancer. *JAMA Netw Open*. Published February 24, 2023.
doi:10.1001/jamanetworkopen.2023.0122

Data
Data available: No
